# Irritable bowel syndrome is concentrated in people with higher educations in Iran: an inequality analysis

**DOI:** 10.4178/epih.e2017005

**Published:** 2017-02-01

**Authors:** Asieh Mansouri, Mostafa Amini Rarani, Mosayeb Fallahi, Iman Alvandi

**Affiliations:** 1 Department of Epidemiology and Biostatistics, School of Public Health, Tehran University of Medical Sciences, Tehran, Iran; 2 Health Management and Economics Research Center, Isfahan University of Medical Sciences, Isfahan, Iran; 3 Shahid Sadoughi University of Medical Sciences, Yazd, Iran

**Keywords:** Irritable bowel syndrome, Socioeconomic factors, Inequality, Mental health, Iran

## Abstract

**OBJECTIVES:**

Like any other health-related disorder, irritable bowel syndrome (IBS) has a differential distribution with respect to socioeconomic factors. This study aimed to estimate and decompose educational inequalities in the prevalence of IBS.

**METHODS:**

Sampling was performed using a multi-stage random cluster sampling approach. The data of 1,850 residents of Kish Island aged 15 years or older were included, and the determinants of IBS were identified using a generalized estimating equation regression model. The concentration index of educational inequality in cases of IBS was estimated and decomposed as the specific inequality index.

**RESULTS:**

The prevalence of IBS in this study was 21.57% (95% confidence interval [CI], 19.69 to 23.44%). The concentration index of IBS was 0.20 (95% CI, 0.14 to 0.26). A multivariable regression model revealed that age, sex, level of education, marital status, anxiety, and poor general health were significant determinants of IBS. In the decomposition analysis, level of education (89.91%), age (−11.99%), and marital status (9.11%) were the three main contributors to IBS inequality. Anxiety and poor general health were the next two contributors to IBS inequality, and were responsible for more than 12% of the total observed inequality.

**CONCLUSIONS:**

The main contributors of IBS inequality were education level, age, and marital status. Given the high percentage of anxious individuals among highly educated, young, single, and divorced people, we can conclude that all contributors to IBS inequality may be partially influenced by psychological factors. Therefore, programs that promote the development of mental health to alleviate the abovementioned inequality in this population are highly warranted.

## INTRODUCTION

Irritable bowel syndrome (IBS) includes a group of functional bowel disorders in which abdominal discomfort or pain is associated with defecation or a change in bowel habits, and with features of disordered defecation [1]. There is a well-established statistically significant heterogeneity in IBS prevalence among various regions of the world. According to a published review, the pooled regional prevalence of IBS was 17.5, 9.6, 7.1, and 5.8% in Latin America, Asia, North America/Europe/Australia/New Zealand, and the Middle East/Africa, respectively [2]. Another study reported an IBS prevalence of 10 to 15% and 5 to 10% in Western and Asian countries, respectively [3]. A systematic review also reported that the prevalence of IBS ranged from 1.1 to 25% in Iran [4]. IBS has various adverse effects on a patient’s social life and work, such as increased absenteeism, reduced quality of life, and substantial medical costs [5].

Like some other health-related disorders, IBS has a diverse distribution with respect to socioeconomic factors. For example, a cohort study showed that the prevalence of IBS was significantly higher in people with a lower household income [6]. Moreover, Andrews et al. [7] reported a decreasing trend of IBS prevalence according to income and education from the lowest to the highest groups. Another study also reported that unemployed individuals were more prone to IBS than employed individuals [8].

Despite the well-established diverse distribution of IBS according to socioeconomic status, the determinants of this diversity have not been identified and interpreted using specific inequality indices. Because of the importance of identifying the characteristics of inequalities in IBS, a decomposition analysis of educational inequality, which was the main aim of the present study, might reveal useful information for policymaking. The results of this study are expected to help to make decisions and design programs for alleviating IBS inequality in the future.

## MATERIALS AND METHODS

We used data from a household survey on IBS that was conducted on Kish Island in 2009. The large number of islanders visiting Kish Hospital with the chief complaint of psychological and gastrointestinal symptoms justified a survey of the prevalence and familial aggregations of IBS occurrence. In this survey, 2020 people in 343 households were interviewed. A multi-stage cluster sampling method was used for selecting the study participants. More details about the sampling scheme have been published elsewhere [9].

The outcome variable in our study was the presence or absence of IBS, which was measured using the Rome II diagnostic criteria for IBS. This is a widely used tool for the diagnosis of functional bowel disorders and functional abdominal pain. The criteria for IBS diagnosis in this tool include abdominal discomfort or pain that has two of the following three features: 1) relief with defecation, 2) onset associated with a change in stool frequency, and 3) onset associated with a change in the stool form or appearance for at least 12 weeks, not necessarily consecutive, in the preceding 12 months [1].

We excluded people aged people aged less than 15 years from the analysis because of the small number of individuals with IBS in this age group (n=1); hence, the study sample size dropped to 1,850. To estimate inequality, the level of education (measured as the number of years of education successfully completed) was determined as a proxy for socioeconomic status.

Age, sex, marital status (single, married, or divorced), occupation (housekeeper, unemployed, office worker, self-employed, retired, or student), poor general health measured using the General Health Questionnaire (GHQ) scale (categories: no, GHQ< 24; yes, GHQ> 24; Ebrahimi et al. [10]), anxiety measured using the Beck Anxiety Inventory (BAI) scale (categories: no, BAI< 22; yes, BAI> 22; Beck et al. [11]), history of gastrointestinal disorders (yes/no), and cigarette smoking status (yes/no) were determined as the study covariates. On the basis of years of education, a new categorical variable referred to as level of education was generated; it had the following categories: primary (< 5 years of education), secondary (5-12 years of education), and postsecondary (>12 years of education).

### Statistical analysis

Stata version 11/SE (StataCorp., College Station, TX, USA) was used for the analysis. Educational inequality in the cases of IBS was measured using the concentration index. The equation formulated by Kakwani et al. [12] was used to estimate the concentration index, which was calculated as twice the covariance of the health-related outcome variable and the fractional rank in the standard living distribution divided by the mean health-related outcome (equation 1). In this formula, *h_i_*, *r_i_*, and *µ* denote the health status of the *i*^th^ individual, the fractional rank of the *i*^th^ individual related to the standard living variable (education in this study), and the average of the outcome variable, respectively. The concentration index varied between −1 and +1, indicating disproportional concentrations of the health outcome among the people with a low or high status of the socioeconomic proxy variable, respectively. The concentration index is 0 when the health outcome has no inequality according to the socioeconomic proxy variable.

(1) $$C=\frac{2}{\mu }\mathrm{cov}(h_{i},r_{i})$$

As our sample consisted of household members, we took into account the cluster sampling effect in the estimation of the concentration index according to the method presented by O’Donnell et al. [13].

### Inequality decomposition

For the decomposition of inequality in the outcome variable, we first identified the determinants of the outcome by using a suitable regression model (equation 2).

(2) $$Y_{i}=a+\sum_{k}\beta_{k}x_{k}+\varepsilon_{i}$$

where *Y_i_*, *β_k_*, and *ε_i_* denote the outcome variable, regression coefficient, and the error term, respectively. In its simplest state (with a continuous outcome variable), this will be a linear regression model.

Given that the outcome in our study was a binary variable (yes/no) and our study participants were clustered in families, we used a generalized estimating equation regression model to identify the determinants of IBS.

After identifying the abovementioned determinants, we decomposed the corresponding concentration index according to the approach introduced by Wagstaff et al. [14]; this approach is presented in equation 3:

(3) $$C=\sum_{k}(\frac{\beta_{k}\bar{x_{k}}}{\mu })C_{k}+\frac{GC_{\varepsilon }}{\mu }\;=C_{y}+\frac{GC_{\varepsilon }}{\mu }$$

In equation (3), ${\bar{x}}_{k}$, *C_k_*, and *GC_ε_* denote the mean for the *k*^th^ determinant, concentration index for the *k*^th^ determinant (defined analogously to the concentration index for the health variable in question), and generalized concentration index for *εi*, respectively. More details about concentration index decomposition have been presented elsewhere [13,14].

All participants signed written informed consent forms. The questionnaires were completely anonymous. The study was approved by the Ethics Committee of Tehran University of Medical Sciences.

## RESULTS

The data of 1,850 participants aged 15 years or more were used in the analysis. The mean± standard deviation of the age and of the years of education was 40.27± 15.00 years and 12.60± 3.37 years, respectively. The characteristics of the participants are presented in Table 1. As shown, most of the participants were young, female, married, and self-employed, and with secondary education. The frequency of people with anxiety, poor general health, history of gastrointestinal disorders, and history of cigarette smoking was remarkable.

Of the sample, 399 people (21.57%; 95% CI, 19.69 to 23.44) had IBS. The frequency of IBS with respect to the exploratory variables is presented in Table 1. As shown in Table 1, IBS was more prevalent among the age group of 26-50 years; females; divorced and unemployed individuals; people with anxiety, poor general health, and a positive history of gastrointestinal disorders; smokers; and people with postsecondary education.

**Figure 1.**
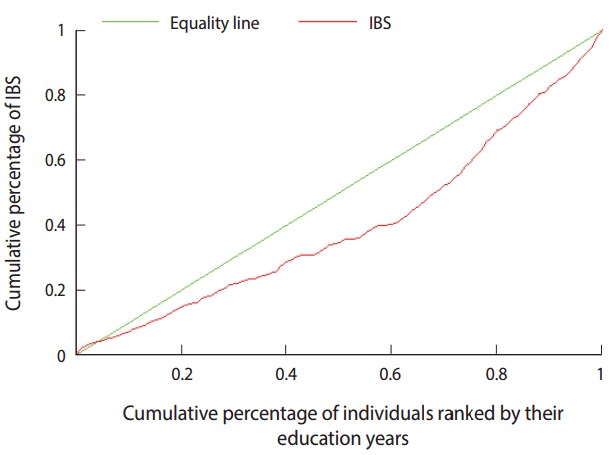
Concentration curve for irritable bowel syndrome (IBS) on Kish Island, 2009.

The concentration index of IBS was 0.20 (95% CI, 0.14 to 0.26). This implies that IBS did not have an equal distribution among people with different levels of education. In other words, persons with IBS were concentrated among people with a relatively high education. Figure 1 depicts the concentration curve for IBS. This curve lies below the equality line, which implies that IBS was more prevalent among people with relatively high education.

The relationship of education with the other variables is presented in Table 2. As shown, the mean of the years of education was higher among people aged 26-50 years, males, single individuals, unemployed individuals, people with anxiety and poor general health, people without a positive history of gastrointestinal disorders, and cigarette smokers. Among these variables, only sex and history of gastrointestinal disorders did not have a statistically significant relationship with education.

We identified the determinants of IBS by using a generalized estimating equation regression model as a primary step for the IBS educational inequality decomposition. We used the forward strategy, introduced by Hosmer & Lemeshow [15], for building the model. A significance level of 0.20 and 0.05 was considered for the univariate and multivariate analysis, respectively. The variables of age, sex, marital status, occupation, history of gastrointestinal disorders, general health status, anxiety, history of cigarette smoking, and years of education were entered in the univariate analysis. Variables presented in Table 3 remained in the final model. We calculated the contribution of the IBS determinants to the corresponding educational inequality by using a decomposition analysis. The results are presented in Table 3. The educational inequality for each IBS determinant is shown in the fourth column along with the corresponding concentration index. For example, the concentration index of the age group of 26-50 years was positive, implying that individuals in this age group were concentrated among people with more years of education. On the other hand, it was negative for the age group of more than 50 years, indicating that individuals in this age group were concentrated among people with fewer years of education. The contribution of each determinant to the educational inequality in cases of IBS is presented in the last column. The main contributors to educational inequality in cases of IBS were education, age, marital status, anxiety, and poor general health (in the order of importance). Other determinants, including sex and a positive history of gastrointestinal disorders, had minor contributions (less than 2% overall).

The frequency of anxiety and poor general health with respect to the main contributors to IBS is presented in Table 4. The frequency of anxiety and poor general health was the highest in people with postsecondary education, people aged 51 years or older, and divorcees as compared to their counterparts (Table 4).

## DISCUSSION

In comparison to some other studies in Iran, this study demonstrated a higher prevalence of IBS [2,16]. This could be attributed to the specific conditions of life of the Kish Islanders. As mentioned before, a remarkable proportion of participants had anxiety, which has been identified as a risk factor for IBS [17]. The islanders are exposed to various stressors such as long working hours and a lack of stable jobs. According to a study, the assignment of most employment with economic and cultural potential to non-natives on Kish Island can cause depression and anxiety in the local people [18]. Another probable cause is diet. Khayyatzadeh et al. [19] reported that dietary patterns were an effective factor for alleviating or aggravating the symptoms of IBS. They implied that a vegetarian diet helped to decrease the risk of IBS in Iranian adults. Although we did not assess the dietary patterns of the participants in this study, given that the island does not have a conducive environment for agriculture, the limited access of residents to adequate amounts of fresh fruits and vegetables could be another explanation for the high prevalence of IBS in this region. In addition, the participants in this study were often family members. Multiple studies have shown that the presence of an individual with IBS in a family significantly increases the risk of IBS in the other family members [20-23]. Therefore, another reason for the high prevalence of IBS in our study could be the statistically significant familial aggregation due to our sampling type.

The concentration index and the concentration curve for educational inequality in cases of IBS demonstrated that IBS was significantly concentrated in people with relatively high education. In the decomposition analysis, most determinants showed a positive contribution to the educational inequality in cases of IBS. This implies that this determinant helped to increase the IBS inequality to disfavor people with relatively high education. A negative contribution, on the other hand, implies that the variable contributed to the alleviation of the IBS inequality in favor of relatively highly educated people.

The contributors of this inequality, from the most to the least important, included education, age, marital status, anxiety, general health status, sex, and a history of gastrointestinal disorders.

As mentioned earlier, the main contributor to this inequality was education. According to some studies, IBS has an inverse relationship with education [7,8,24,25]; however, we observed the opposite. The prevalence and risk of IBS was higher in people with postsecondary education than in less-educated people. Ibrahim et al. [26] found that IBS was more prevalent among nurses with a level of education of university or above than among their counterparts. They indicated elsewhere that in medical students, IBS prevalence had an increasing trend according to the academic year of the students [27]. Costanian et al. [28] observed this finding as well. According to Roohafza et al. [29], educational concerns are an important group of life stressors, which along with job-related stressors could significantly predict IBS occurrence. There do not seem to be a sufficient number of suitable occupations for highly educated people in places like Kish Island. Therefore, efforts to find suitable jobs and perhaps a lack of sufficient income can make people with postsecondary education prone to anxiety. We observed that the frequency of anxiety had a similar trend to that of IBS with respect to the level of education. Therefore, we concluded that highly educated participants in our study had more anxiety, which is an established risk factor for IBS [17,29]. Accordingly, an important step for alleviating the educational inequality in IBS would seem to be to pay special attention to the identification and elimination of the sources of stress in people with a high level of education.

The next contributor of IBS inequality in this study was age. Qureshi et al. [30] stated that IBS did not have any statistically significant associations with age groups. However, we observed that the risk of IBS was significantly higher in the age groups of 26-50 years and more than 50 years than in the reference age group of 15-25 years. Hungin et al. [31] found that the prevalence of IBS was higher among people aged 25-54 years than among other age groups. In another study, the risk of IBS was higher in people older than 30 years than in individuals aged 30 years or less [26]. In contrast, some studies reported that the risk of IBS was significantly higher in the youth. For example, Costanian et al. [28] presented an adjusted odds ratio of 1.89 for people aged 18-22 years versus people aged more than 22 years. In another study, the prevalence of IBS was observed to be the highest in the age group of 21-30 years, with a decreasing trend from this group to the age group of more than 60 years [32]. Han et al. [25] suggested that the relatively high prevalence of IBS in young people may be due to psychological factors, such as stress related to studies, finding jobs, economic status, or marriage.

The next contributor to IBS inequality was marital status. The prevalence and risk of IBS were higher in single and divorced people than in their married counterparts. This finding is consistent with the results of other studies [7,24]. According to Abdulmajeed et al. [24], a higher prevalence of IBS in unmarried than in married people may be due to more responsibilities and stressors in these groups. However, Han et al. [25] found a contrasting finding, which could be attributed to marital problems.

Anxiety and poor general health were the next two contributors to IBS inequality that were responsible for more than 12% of the observed inequality. This implies that these factors contributed to an increased concentration of IBS in people with higher education. As the relationship between mental health and IBS has been confirmed by multiple studies [30], we recommend setting up programs with the aim of reducing anxiety and promoting mental health in Kish Islanders, particularly the highly educated, to help decrease the educational inequality in cases of IBS.

A minor contributor to IBS inequality was sex. Being female was associated with a higher risk of IBS. This finding has been reported by multiple studies [30,32-34]. Pan et al. [33] attributed this sex difference to female hormones as a result of the declining incidence of IBS in females after menopause. Chang & Heitkemper [34] reported that sex-related differences in gastrointestinal transit time, visceral sensitivity, central nervous system pain processing, neuroendocrine, autonomic nervous system, and stress reactivity can justify the predomination of IBS in females. In contrast, Farzaneh et al. [8] stated this difference to be a result of a selection bias due to a higher likelihood of seeking healthcare in females.

A positive history of gastrointestinal disorders was the most minor contributor to IBS inequality in this study. Ansari et al. [35] reported that people with ulcerative colitis had a higher risk of developing IBS than healthy controls. According to a review article, a conclusive relationship was found between previous bowel disorders and IBS occurrence [36].

Occupation had a statistically significant relationship with IBS in the univariate analysis. As mentioned earlier, the prevalence of IBS was higher in unemployed people. Modabbernia et al. [17] found a significantly higher frequency of IBS in jobless people than in others. Farzaneh et al. [8] reported that lower income and severe psychological distress in unemployed people made them more prone to having IBS than employed individuals.

Although IBS prevalence was significantly higher in smokers, we did not find any statistically significant relationship between smoking and IBS in the multivariable analysis. This finding is consistent with the reports of the previous studies [25,32].

### Strengths and limitations

To the best of our knowledge, this is the first study that conducted an inequality analysis of IBS. In this study, a large-scale population-based sampling enabled us to obtain highly precise results. However, this study had some limitations. As mentioned, the proxy variable of socioeconomic status was needed to estimate and decompose inequality. In this study, proxy variables for the standard of living, such as income and wealth status, were not measured. People’s reluctance to disclose information related to their income as a result of concerns related to tax payments and income fluctuations due to the seasonality of jobs were the main obstacles to asking questions about income in this region. Therefore, education was the only variable associated with socioeconomic status in our data. Education alone may not be a comprehensive indicator of socioeconomic status. Therefore, we suggest an assessment of IBS inequality with respect to other indicators of socioeconomic status, particularly wealth, in the future.

As another weakness, the study participants were family members. Therefore, our estimations may slightly differ from those of studies with independent participants. On the other hand, Yiengprugsawan et al. [37] demonstrated that generalized linear models with a binomial distribution and an identity link function are a suitable choice for decomposition analysis when the outcome variable is binary because of the estimates of determinants that are unchangeable with respect to the choice of reference group. However, we could not use this model for the decomposition analysis as a result of the correlated outcome and the failure of the independence assumption. Therefore, we recommend an assessment of IBS inequalities in an independent, large sample in future studies.

In conclusion, we showed that IBS demonstrated statistically significant educational inequality, with a disproportional concentration in highly educated persons. The most important contributors to this inequality were education, age, and marital status. Given the high percentage of anxious individuals among highly educated, young, single, and divorced people, we can conclude that all contributors to IBS inequality may be partially influenced by psychological factors. We recommend developing anxiety reduction and mental health promotion programs for the people of Kish Island as a helpful solution to alleviate the unequal distribution of IBS.

## Figures and Tables

**Table 1.** Socio-demographic characteristics of Kish residents aged 15 years and above and prevalence of irritable bowel syndrome (IBS) in terms of these characteristics in 2009

Variable	n (%)	IBS distribution		
		n (%)	p-value^1^
Age (yr)			
15-25	395 (21.35)	65 (16.46)	<0.001
26-50	989 (53.46)	230 (23.26)	
51 or older	466 (25.19)	104 (22.32)	
Sex			
Male	882 (47.68)	140 (15.87)	<0.001
Female	952 (51.46)	258 (27.10)	
Unknown	16 (0.86)	1 (6.25)	
Marital status			
Single	544(29.41)	146 (26.84)	<0.001
Married	1,231 (66.54)	227 (18.44)	
Divorced	75 (4.05)	26 (34.67)	
Job status			
Housekeeper	329(17.78)	83 (25.23)	0.004
Unemployed	267(14.43)	73 (27.34)	
Office worker	303(16.38)	57 (18.81)		
Self-employed	564 (30.49)	117 (20.74)	
Retired	93 (5.03)	20 (21.51)	
Student (school or university)	263(14.22)	38 (14.45)	
Unknown	31 (1.68)	11 (35.48)	
Anxiety (BAI ≥22)			
Yes	218(11.78)	123 (56.42)	<0.001
No	1,572 (84.97)	266 (16.92)	
Unknown	60 (3.24)	10 (16.67)	
Poor general health (GHQ ≥24)			
Yes	121 (6.54)	83 (68.60)	<0.001
No	1,668 (90.16)	306 (18.53)	
Unknown	61 (3.30)	10 (16.39)	
History of gastrointestinal disorders			
Yes	214(11.57)	107 (50.00)	<0.001
No	1632 (88.22)	292 (18.89)	
Unknown	4 (0.22)	0 (0.00)	
Cigarette smoking			
Yes	463 (25.03)	115 (24.84)	0.05
No	1,387(74.97)	284 (20.48)	
Level of education (yr)			
Primary (≤5 )	103 (5.57)	19 (18.45)	<0.001
Secondary (6-12)	1,010 (54.59)	142 (14.06)	
Academic (>12)	737 (39.84)	238 (32.29)	

BAI, Beck Anxiety Inventory score; GHQ, General Heath Questionnaire score.

1 Chi-square test.

**Table 2.** Relationship of education with other variables in Kish residents aged 15 years and above in 2009

Variable	Mean	SD	p-value
Age (yr)			
15-25	13.13	2.31	<0.001^1^
26-50	13.49	2.66	
51 or older	10.28	4.28	
Sex			
Male	12.67	3.23	0.44^2^
Female	12.55	3.51	
Marital status			
Single	13.61	2.56	<0.001^1^
Married	12.23	3.47	
Divorced	11.51	4.87	
Job status			
Housekeeper	12.20	3.45	<0.001^1^
Unemployed	13.83	2.69	
Office worker	12.84	2.92	
Self-employed	12.59	3.28	
Retired	9.45	4.73	
Student (school or university)	12.55	3.28	
Anxiety (BAI ≥22)			
Yes	13.32	3.28	0.02^2^
No	12.80	2.95	
Poor general health (GHQ ≥24)			
Yes	13.62	3.34	0.004^2^
No	12.81	2.96	
History of gastrointestinal disorders			
Yes	12.30	4.11	0.16^2^
No	12.65	3.26	
Cigarette smoking			
Yes	13.02	3.52	0.002^2^
No	12.47	3.31	

SD, standard deviation; BAI, Beck Anxiety Inventory score; GHQ, General Heath Questionnaire score.

1 One-way analysis of variance test.

2 Independent t-test.

**Table 3.** Adjusted determinants of irritable bowel syndrome and decomposition of its concentration index in Kish residents aged 15 years and above in 2009

	Coefficient	Mean	Elasticity	CI	Contribution (%)
Age (yr)					
15-25	Reference				
26-50	0.762	0.53	-0.295	0.133	19.10
51 or older	1.060	0.25	-0.193	-0.331	-31.09
Total					-11.99
Sex					
Male	Reference				
Female	0.793	0.52	-0.298	0.003	0.49
Marital status					
Married	Reference				
Single	0.563	0.29	-0.120	0.173	10.09
Divorced	0.742	0.04	-0.022	-0.092	-0.98
Total					9.11
Anxiety (BAI ≥22)					
No	Reference				
Yes	1.206	0.12	-0.106	0.120	6.22
Poor general health (GHQ ≥24)					
No	Reference				
Yes	1.605	0.07	-0.078	0.152	5.83
History of gastrointestinal disorders					
No	Reference				
Yes	1.393	0.12	-0.117	0.007	0.43
Level of education (yr)					
Primary (≤5)	-1.335	0.06	0.054	-0.943	24.64
Secondary (6-12)	-0.993	0.55	0.393	-0.342	65.27
Academic (>12)	Reference				
Total					89.91

CI, concentration index; BAI, Beck Anxiety Inventory score; GHQ, General Heath Questionnaire score.

**Table 4.** Number and percentage of people with anxiety and poor general health by the 3 main contributors to IBS inequality among Kish residents aged 15 years and above in 2009

	Total (n)	Anxiety (BAI ≥ 22)			Poor general health (GHQ ≥ 24)		
		n	%	p-value	n	%	p-value
Level of education (yr)							
Primary (≤5)	103	9	13.64	0.001	5	7.58	0.001
Secondary (6-12)	1,010	95	9.62		48	4.86	
Academic (>12)	737	114	15.49		68	9.25	
Age (yr)							
15-25	395	39	10.05	0.14	23	5.94	0.12
26-50	989	117	11.98		60	6.14	
51 or older	466	62	14.59		38	8.94	
Marital status							
Married	544	122	10.23	0.002	62	5.20	0.001
Single	1,231	87	16.23		51	9.51	
Divorced	75	9	7.40		8	13.11	

IBS, iritable bowel syndrome; BAI, Beck Anxiety Inventory score; GHQ, General Heath Questionnaire score.
